# Sheep Rumen Fermentation Characteristics Affected by Feeding Frequency and Feeding Level When Fed Fresh Forage

**DOI:** 10.3390/ani10010007

**Published:** 2019-12-19

**Authors:** Xuezhao Sun, Ao Chen, David Pacheco, Simone O. Hoskin, Dongwen Luo

**Affiliations:** 1 The Innovation Centre of Ruminant Precision Nutrition and Smart and Ecological Farming, Jilin Agricultural Science and Technology University, Jilin 132109, China; 2 Jilin Inter-regional Cooperation Centre for the Scientific and Technological Innovation of Ruminant Precision Nutrition and Smart and Ecological Farming, Jilin 132109, China; 3 Faculty of Agriculture and Life Science, Lincoln University, Lincoln 7647, New Zealand; archeraochen@gmail.com; 4 Grasslands Research Centre, AgResearch Limited, Private Bag 11008, Palmerston North 4442, New Zealand; david.pacheco@agresearch.co.nz (D.P.); dongwen.luo@agresearch.co.nz (D.L.); 5 Fiber Fresh Feeds Limited, RD2, Reporoa 3083, New Zealand; simone.hoskin@fiber-fresh.com

**Keywords:** rumen fermentation, feeding frequency, feeding level, fresh forage, sheep

## Abstract

**Simple Summary:**

Ruminant production relies on nutrients obtained from feed fermentation in the rumen. In grazing ruminant production systems, fresh forage is the major or sole source of feed. Feeding frequency and feeding level are two important factors affecting rumen fermentation. This study investigated how these factors affect the fermentation of fresh forage in the rumen for in-depth evaluation of the nutritional value of two types of forages and for better understanding of their digestion by ruminants. To achieve the objectives of the study, fresh chicory or perennial ryegrass was fed to sheep hourly or twice-daily at low or high feeding levels. The results indicated that rumen fermentation was affected by forage species, feeding frequency, feeding level and their interactions and the differences in rumen fermentation were more apparent when feeding was performed hourly rather than twice-daily. This study highlighted the importance of feeding frequency on manipulating sheep ruminal metabolism when fed fresh forage.

**Abstract:**

Feeding frequency and feeding level are two important factors affecting rumen fermentation characteristics, but few studies on these have been conducted on fresh forage. Eight rumen-fistulated sheep were fed either fresh chicory or perennial ryegrass hourly in the first period (d 14 to 21) of the experiment and twice-daily in the second period (d 22 to 27) at 1.3 or 2.2 times the requirement of metabolizable energy for maintenance. When fed hourly, but not twice-daily, rumen fluid pH value was affected by forage species and feeding level. The total concentrations of short-chain fatty acid (SCFA) were similar at both feeding levels when fed chicory hourly, but they were greater at the higher feeding level in comparison with the lower feeding level when fed perennial ryegrass. However, forage species and feeding level did not affect rumen fluid total SCFA concentration when sheep were fed twice-daily. Therefore, rumen fermentation characteristics were affected by forage species, feeding frequency, feeding level and their interactions and the differences in fermentation characteristics were more apparent when feeding was performed hourly rather than twice-daily. This study highlighted the importance of feeding frequency on manipulating sheep ruminal metabolism when fed fresh forage.

## 1. Introduction

Animal nutrition studies focuses on how animals respond to different feeds and feeding technologies. Rumen fermentation characteristics are considered to be effective indicators in the evaluation of feeds and feeding for ruminants [1]. However, the once-or twice-daily feeding regime, which results in large fluctuations in rumen fermentation, as well as variable nutrient flows for utilization by ruminants, has often been employed in previous studies [2,3].

Feeding frequency affects many aspects of ruminants, including rumen fermentation function [4], dry matter voluntary intake and feed digestion [5], feces and urine excretion [6], and animal performance [7,8] and behavior [9]. Herbs, such as chicory, are increasingly used as alternative forage species for dairy and meat production either in their monocultures or mixtures with legumes [10,11]. Previous feeding experiments evaluating these novel forage species were mostly conducted under a two meals a day feeding regime with feeding levels around maintenance metabolizable energy (ME) requirements [12,13]. However, to our knowledge, little information is available on how feeding frequency and feeding level affect sheep rumen fermentation when fed different fresh forage diets. Such knowledge is necessary to improve the understanding of rumen function as well as the interpretation of research findings because of the potential interactions between those factors. This information may also be helpful in animal production practice, as changes in grazing management or/and feeding regime could easily manipulate feeding frequency and feeding level in practical farming situations [14]. Therefore, the objective was to define how feeding level and forage species would affect sheep rumen fermentation characteristics under different feeding frequencies.

## 2. Materials and Methods 

### 2.1. Experimental Design and Animals

The study was performed at AgResearch Grasslands Research Centre (Palmerston North, New Zealand) during autumn under the approval of the AgResearch Grasslands Animal Ethics Committee. The study consisted of two periods in which eight rumen-fistulated Romney whether sheep (approximately 20 months old and 48 ± 2.9 kg in weight) were fed the experimental diets. Forage species and feeding level were two experimental factors separately analyzed in a 2 × 2 factorial design for each period. Forage diets included chicory and perennial ryegrass at two feeding levels: 1.3 and 2.2 × maintenance ME, recommended by the Australian Agricultural Council [15]. The experimental animals were randomly allocated to two groups with each group of four grazed on pure chicory or perennial ryegrass pastures for 7 d. Then they were held in individual pens for further acclimation for 6 d. During the indoor acclimation period, half of the animals were fed at a low feeding level, the other half at a high feeding level. Then the sheep were transferred into metabolic crates from d 14 to 27 for measurements. The feeding frequency was set as ‘hourly’ (high feeding frequency, 24 meals a day) in the first period of the experiment from d 14 to 21 and ‘twice-daily’ (low feeding frequency, 2 meals a day) in the second period from d 22 to 27. Rumen fluid samples were taken on d 21 for the 24 meals a day feeding frequency and on d 27 for the 2 meals a day feeding frequency.

### 2.2. Forage and Feeding

Chicory (*Cichorium intybus* cv. Choice) had regrown for 6 wk before grazing or indoor feeding and was about 50 cm high above ground level; perennial ryegrass (*Lolium perenne* cv. Quartet) had regrown for 4 wk and was about 35 cm above ground level. For indoor feeding, herbage was harvested between 10:00 and 12:00 h using a sickle bar mower and stored at 4 °C before feeding to sheep [16]. The chemical compositions and ME of chicory and perennial ryegrass herbage were evaluated by near infrared reflectance spectroscopy (NRIS, FeedTECH) as described by Sun et al. [17] and presented in Table 1. 

During the grazing adaptation period, herbage was provided generously. When fed indoors, sheep received the designated feeding levels, which was either 1.3 or 2.2 × maintenance ME. The amount of forage provided was calculated based on the forage ME content. Sheep were provided 24 meals on the hour or 2 equal meals at 09:00 and 16:30 h every day using automated overhead belt-feeders. Water was provided *ad libitum*.

### 2.3. Rumen Fermentation Parameters

On each sampling day, rumen fluid (~20 mL) was sampled via the rumen fistula from the dorsal sac of the rumen using a 100 mL syringe mounted with a 30 cm long plastic tube at 09:00 (before feeding), 10:00, 11:00, 12:00, 13:00 and 15:00 h. Immediately after sampling, pH values were measured using a PHM210 standard pH meter (Radiometer Analytical, 72 Rue d’Alsace, 69627 Villeurbanne Cedex, Lyon, France). Collected rumen samples were kept over ice, subsampled (1.8 mL) within 0.5 h and then treated as described by Sun et al. [16]. The subsamples were centrifuged (20,000 *g* for 5 min at 4 °C) and the supernatant (0.9 mL) was mixed with an acid solution (0.1 mL) containing orthophosphoric acid (200 µL/mL) and 2-ethyl butyric acid (20 mM). The acid-treated samples were stored at−20 °C until needed. Before analysis, the stored samples were thawed in a fridge overnight and centrifuged again using the same method as described by Sun et al. [16] to obtain supernatant for the determination of ammonia and short-chain fatty acid (SCFA) concentrations. Ammonia concentrations were determined using the nitroprusside method of Weatherburn [18] and SCFA concentrations using gas chromatography [16].

### 2.4. Statistical Analysis

Statistical analyses were conducted using a repeated measurement ANOVA, with the power model as correlation within sheep across time, using REML (GenStat, v17.0) [19]. Forage species, feeding level and their interactions were included in the model as fixed effects and animal as a random effect. In this experimental design, comparisons between feeding frequencies were not feasible, because of the confounding between feeding frequency and experimental period. As a result, statistical analyses for the two feeding frequencies were separately conducted.

## 3. Results

The chemical composition of chicory and perennial ryegrass herbage is summarized in Table 1. Compared with perennial ryegrass, chicory had less crude protein and neutral detergent fiber, and more water-soluble carbohydrates and pectin.

### 3.1. pH Value

**Figure 1:**
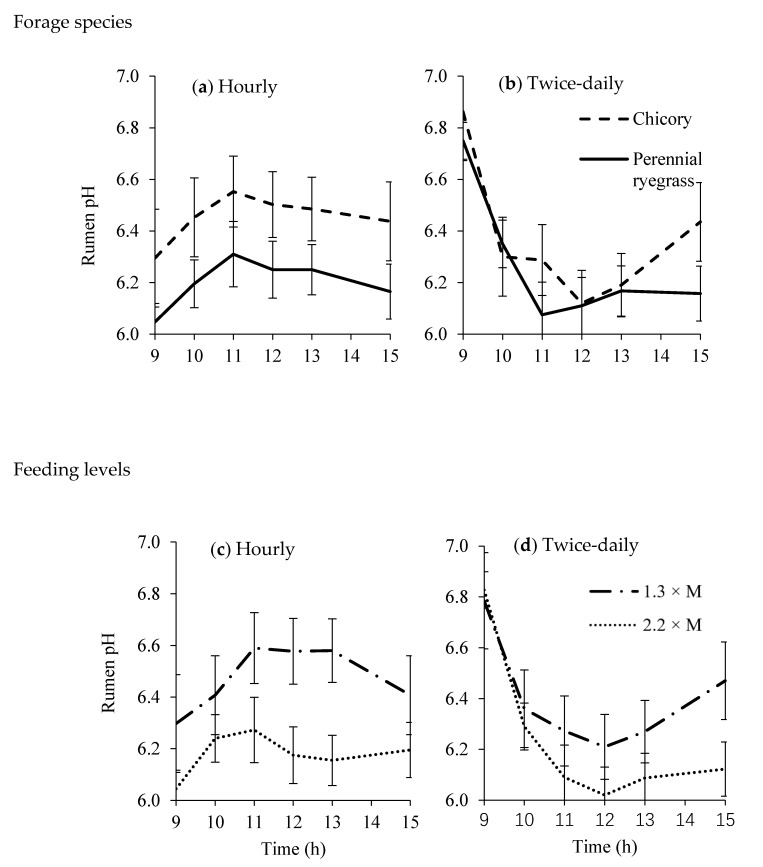
Rumen fluid pH values of sheep fed chicory or perennial ryegrass at 1.3 or 2.2 times maintenance metabolizable energy requirement under hourly (**a**,**c**) or twice-daily (**b**,**d**) feeding frequency (n = 4). bar, SEM.

Overall, there was less variation in rumen fluid pH value for the hourly feeding treatment than for twice-daily feeding treatment (Figure 1). The rumen fluid pH value was higher for sheep fed hourly with chicory than fed with perennial ryegrass (*p* = 0.073; Figure 1a). No significant differences were detected in rumen fluid pH values between the two forage species when sheep were fed twice-daily (*p* = 0.350; Figure 1b) except at 15:00 h. The pH value of rumen fluid was higher in sheep fed a low feeding level compared to a high feeding level (6.48 *versus* 6.18; *p* = 0.070; Figure 1c) when they were fed hourly, but no differences were detected when sheep were fed twice-daily, except at 15:00 h (*p* = 0.349; Figure 1d). The interactions between forage species and feeding level were not significant when fed hourly (*p* = 0.401) or twice-daily (*p* = 0.876).

### 3.2. Ammonia

**Figure 2:**
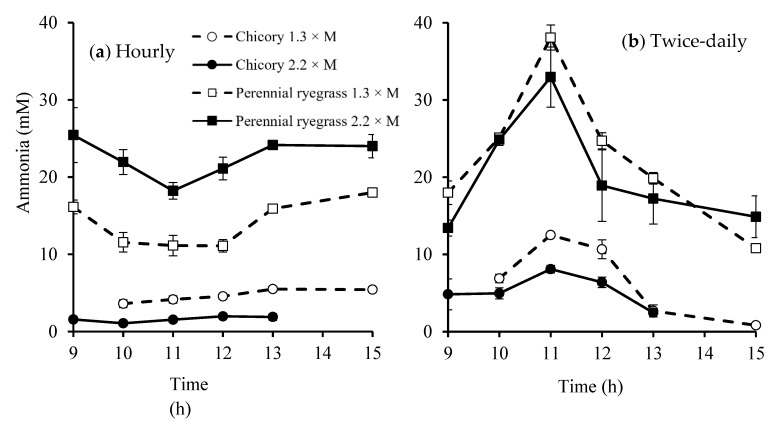
Rumen fluid ammonia concentrations of sheep fed chicory or perennial ryegrass at 1.3 or 2.2 times maintenance metabolizable energy requirement under hourly (**a**) or twice-daily (**b**) feeding frequency (n = 2). bar, SEM.

Sheep fed perennial ryegrass always had greater rumen fluid ammonia concentrations than those fed chicory (28 *versus* 8 mM, *p* = 0.001). Rumen fluid ammonia concentrations remained relatively constant when fed hourly. The interaction was significant (*p* = 0.005; Figure 2a) for rumen fluid ammonia concentration between forage species and feeding level. Specifically, at the low feeding level, rumen fluid ammonia concentrations were lower when sheep were fed chicory (5 mM) than perennial ryegrass (18 mM) while this difference increased to 2 *versus* 27 mM for chicory and perennial ryegrass at the high feeding level. 

In the twice-daily feeding frequency, the concentration of rumen fluid ammonia peaked at around 11:00 h (2 h after morning feeding; Figure 2b) and the peak was higher for perennial ryegrass (41 mM) than chicory (14 mM; *p* < 0.001). Feeding level did not affect rumen fluid ammonia concentration significantly (*p* = 0.579) and no interactions were found between feeding level and forage species either (*p* = 0.337).

### 3.3. SCFA

**Figure 3:**
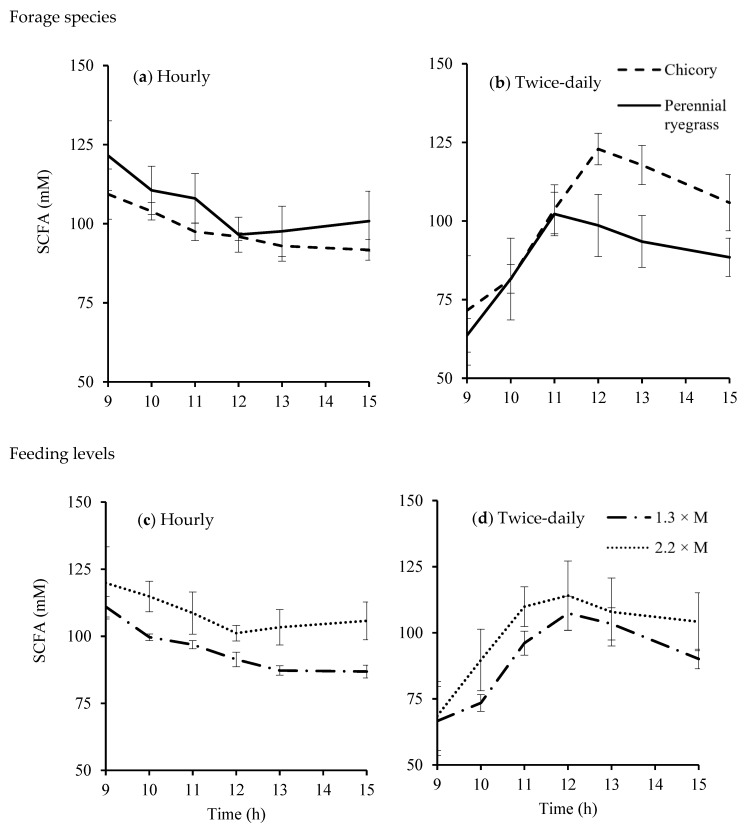
Short chain fatty acid (SCFA) concentrations in rumen fluid of sheep fed chicory or perennial ryegrass at 1.3 or 2.2 times maintenance metabolizable energy requirement under hourly (**a**,**c**) or twice-daily (**b**,**d**) feeding frequency (n = 4). bar, SEM.

Feeding frequency significantly changed total SCFA concentrations (Figure 3) but did not change the molar proportions of individual SCFAs. When sheep were fed hourly, feeding level affected the total SCFA concentrations (*p* = 0.005) and interacted with forage species (*p* = 0.005). The total SCFA concentrations of rumen fluid of sheep fed chicory were similar (99 mM) for the two feeding levels. In contrast, the total SCFA concentrations of rumen fluid of sheep fed perennial ryegrass were greater at the higher feeding level (120 *versus* 92 mM). However, the effects of feeding level and forage species on total SCFA concentration as well as their interaction were not significant when sheep were fed twice-daily. 

Forage species and feeding level did not affect the molar proportions of acetate and propionate. Compared with chicory, the proportion of *n*-butyrate was lower when sheep were fed perennial ryegrass under both feeding frequencies (*p* < 0.001).

## 4. Discussion

In this study, the novelty is the investigation of how feeding frequency and feeding level affect sheep rumen fermentation profiles when fed fresh forage diets. The use of automated overhead belt-feeders, which could control the meal offering amount and frequency, has been used since the 1960s [20] to minimize diurnal fluctuation in digestive function. Since then, high feeding frequency has been widely implemented in animal experiments to steady the nutrient flux for digestion [21] or to potentially manipulate ruminal metabolism [1,22]. Despite the published research providing evidence that feeding frequency affects ruminal metabolism or animal performance [22,23], reports about fresh forage, especially herbs, such as chicory, are relatively scarce.

Generally, diurnal variations of rumen fermentation parameters were less for sheep fed fresh forage for 24 meals a day than 2 meals a day. A previous study showed that a greater feeding frequency (12 *versus* 2 meals a day) led to a reduced variation in the pH value of rumen fluid when cows were fed a 60% pelleted concentrate and 40% chopped alfalfa hay diet [1]. Additionally, a smaller increase in daily feeding frequency from 2 to 4 meals decreased the diurnal variation of both rumen fluid ammonia concentration and pH values [24]. Increasing the concentrate feeding from 2 to 12 times per day in steers resulted in decreased rumen fluid ammonia concentration and apparent digestibility of the diet containing switchgrass hay and supplements at the ratio of 0.6 to 0.4 [25]. Therefore, feeding frequency has a modulatory effect on rumen conditions over a large range of ruminant species and diets. In this study, a greater feeding frequency reduced the variation in rumen fluid pH values, ammonia concentration and total SCFA concentration over time, regardless of the forage species or the feeding level. 

In this study, rumen fluid ammonia concentrations were lower when sheep were fed chicory, compared with perennial ryegrass. These differences in ammonia concentrations in these studies are probably due to a lower crude protein content of chicory (11%) than perennial ryegrass (20%; Table 1). This is consistent with a previous study [26], where lower ammonia concentrations were found when sheep were fed pure chicory, compared to pure perennial ryegrass. 

This study found that the effects of feeding level and forage species and their interactions on sheep rumen fermentation characteristics, such as rumen fluid pH value and ammonia and total SCFA concentrations, were more likely to be significant when sheep were fed more frequently. This result may result from improved statistical power as the variation of fermentation parameters was lowered. Therefore, it is necessary to consider the frequency of feeding used in a study when interpreting research results.

In addition to indoor feeding conditions, the effect of feeding frequency on rumen fermentation is also important in order to interpret results for grazing studies. For example, the understanding of feeding frequency effects on aspects of rumen function may be helpful for optimizing pasture management in grazing conditions. However, there are relatively few studies focusing on rumen fermentation parameters of grazing ruminants using rumen-fistulated or by stomach-tubing intact animals [27]. In pasture-based livestock production systems, there are various feeding regimes, such as rotational and set stocking grazing, in which the feeding frequency varies. For example, under rotational grazing on dairy farms, fresh pasture breaks are allocated to cows once or twice a day [28] and the within-day portable electric fencings (e.g., TechnoGrazingTM systems) are used in beef farming systems [29]. In these systems, larger feeding bouts would resemble the conditions observed with twice-daily feeding. In contrast, other grazing management such as set stocking grazing used more frequently in sheep farming system would lead to a different grazing pattern. Thus, any future study should consider the effect of feeding frequency on ruminal function to optimize grazing management for better animal performance and reduced environmental impact from animals [30].

## 5. Conclusions

Higher feeding frequency reduced the variation in rumen fermentation parameters over time. A greater feeding frequency and/or a higher feeding level was more likely to be able to detect the effect of forage species on rumen fermentation. This study highlighted the importance of feeding frequency and/or feeding level on manipulating sheep ruminal metabolism when fresh forage diets were fed.

## Figures and Tables

**Table 1:** Chemical composition (g/kg dry matter) of chicory and perennial ryegrass.

Item	Chicory	Perennial Ryegrass
Ash	196	127
Crude protein	114	197
Water-soluble carbohydrates	153	114
Pectin	75	10
Neutral detergent fibre	239	423
Acid detergent fibre	188	218
Hemicellulose	51	204
Cellulose	106	191
